# Identifying foreign language learning burnout: latent profiles, cutoff points, and an explainable web-based calculator

**DOI:** 10.3389/fpsyg.2026.1836626

**Published:** 2026-06-17

**Authors:** Nuoyi She, Xu Chen, Qiang Wan, Kun Zhao, Jiongming Liu, Yanru Li

**Affiliations:** 1 School of English Studies, Xi’an International Studies University, Xi’an, China; 2 Department of Lifelong Education, Graduate School, Hanseo University, Seosan-si, Republic of Korea; 3 Department of Physical Education, Shaanxi Polytechnic University, Xianyang, China; 4 Graduate School of Physical Education, Myongji University, Yongin, Republic of Korea; 5 School of Teacher Development, Shaanxi Normal University, Xi’an, China

**Keywords:** burnout, emotional exhaustion, foreign language learning burnout, latent profile analysis, machine learning, ROC analysis

## Abstract

**Background:**

Foreign language learning burnout (FLLB) is prevalent among English as a foreign language learners, which adversely affects students’ academic performance and mental health. However, the clear cutoff point and individualized risk prediction tools for FLLB remain lacking, limiting its application in practical identification and assessment.

**Methods:**

This cross-sectional study recruited 1,343 Chinese secondary school students and collected data on FLLB, academic stress, engagement, teacher affective support, foreign language enjoyment, English achievement, and demographic characteristics. Latent profile analysis (LPA) was used to identify FLLB risk profiles. The cutoff point was determined using receiver operating characteristic curve (ROC) analysis. Six machine learning models were compared, and their generalizability was evaluated in an external validation set. The best-performing model was selected, interpreted with Shapley additive explanation (SHAP) analysis, and deployed as a web-based calculator.

**Results:**

LPA indicated that 14.2% of students belonged to the FLLB high-risk group. The optimal cutoff point was determined as: Exhaustion ≥14, Cynicism ≥3, and Reduced Efficacy ≥3. Logistic regression performed best, with AUC values of 0.903 (internal test set) and 0.813 (external validation set). SHAP analysis revealed that academic stress and foreign language enjoyment were key predictors.

**Conclusion:**

This study determined an operational cutoff point for FLLB. A well-performing risk prediction model was then developed and validated, which we subsequently deployed as a web-based calculator. The tool reports FLLB risk probabilities and visualizes the direction and relative contribution of key predictors, thereby providing a reference for efficient FLLB risk screening and subsequent targeted learning support and psycho-educational services.

## 1 Introduction

Foreign language learning burnout (FLLB) refers to a lasting adverse affective state that students experience in their foreign language studies and classroom activities, mainly manifested as exhaustion, cynicism, and reduced efficacy (Li et al., 2024). In foreign language learning contexts, learners often face linguistic, cultural, and psychological barriers (Wu et al., 2024a) and often experience anxiety and worry during language input and output (Ma et al., 2025; Yu et al., 2022), which makes them more susceptible to burnout. In China, where high-stakes English examinations strongly shape educational opportunities and employment prospects, students carry additional pressure (Hu and Schaufeli, 2009). Studies show that about 54.9% of Chinese secondary school students suffer from burnout (Zhang et al., 2013; Zhu et al., 2023). Burnout diminishes feelings of personal accomplishment, undermines learning motivation, and consequently hinders academic performance (Madigan and Curran, 2021; Rehman et al., 2020). Such psychological distress may also lead to physical problems, including migraines, sleep disorders, and severe injuries (Salvagioni et al., 2017). Therefore, early identification for FLLB risk is of substantial practical value.

Guided by the demand-resource model of burnout (Demerouti et al., 2001) and the broaden-and-build theory (Fredrickson, 2004), previous studies have confirmed associations between burnout and academic stress, engagement, foreign language enjoyment, teacher affective support, and academic achievement (Olson et al., 2025; Paloș et al., 2019; Song, 2024; Wu et al., 2024b; Wu et al., 2023). However, most evidence remains variable centered and cannot fully capture heterogeneity across subgroups. Because FLLB is multidimensional and context-dependent, a single total score may obscure different combinations of exhaustion, cynicism, and reduced efficacy. Latent profile analysis (LPA) can identify distinct latent subgroups based on continuous variables (Spurk et al., 2020), making it more suitable for revealing heterogeneity in burnout. Existing LPA-based studies on burnout have mainly focused on class identification and profile description (Chirkowska-Smolak et al., 2023; Tomaszek et al., 2024), with relatively few studies further translating latent profiles into operational screening criteria. The FLLB scale developed by Li et al. (2024) provides a valuable measurement tool for this field, but it does not provide contextualized and validated cutoff points for identifying high-risk groups. In addition, in the absence of an independent diagnostic gold standard or clinical interviews, high-risk latent classes identified by LPA may serve as an operational reference classification for receiver operating characteristic curve (ROC) analysis to determine screening thresholds for scales (Peng et al., 2023; van Smeden et al., 2014). Therefore, integrating LPA with ROC analysis may provide a feasible approach for determining operational cutoff points for FLLB (Bányai et al., 2017; Li et al., 2020).

In addition, individualized risk prediction remains insufficient in FLLB research. Adolescent mental health services are often constrained by shortages of professionals, limited service accessibility, and insufficient implementation resources (Galagali and Brooks, 2020). Students may also face barriers in recognizing their own psychological distress and seeking support (Gulliver et al., 2010). These practical challenges highlight the applied value of a concise, easily implementable FLLB risk identification tool. Machine learning algorithms can incorporate multiple burnout-related variables into a unified predictive framework to estimate individual-level risk probabilities (Moons et al., 2015; Van Zyl-Cillié et al., 2024). Therefore, this study aims to establish an individualized risk identification framework for FLLB. Specifically, we apply LPA to real underlying FLLB patterns. Second, we determine the cutoff point for FLLB using ROC analysis. Finally, a machine learning model was developed and validated for prediction, which provides individualized Shapley additive explanation (SHAP) explanations. We further deploy the final model as a web-based calculator, which can generate real-time risk probabilities and visualizes key predictors, thereby supporting early screening and assessment for FLLB among English as a foreign language (EFL) learners in real-world settings.

## 2 Materials and methods

### 2.1 Study design

This study used an observational cross-sectional design and conducted model development and validation for identifying students at high risk of FLLB. The data were collected from a multisite school survey conducted during the same period. Samples from three schools were used as the development set for model development. Samples from another school were used as the external validation set to evaluate model performance. This school included both junior and senior secondary students, covering the two main educational stages of the target population in this study, namely junior and senior secondary EFL students. Data preprocessing was conducted separately for the development and validation sets. Therefore, according to the TRIPOD classification, this study was classified as a Type 2b study. The study followed the STROBE (Supplementary File 1) and TRIPOD (Supplementary File 2) reporting guidelines.

**Figure 1:**
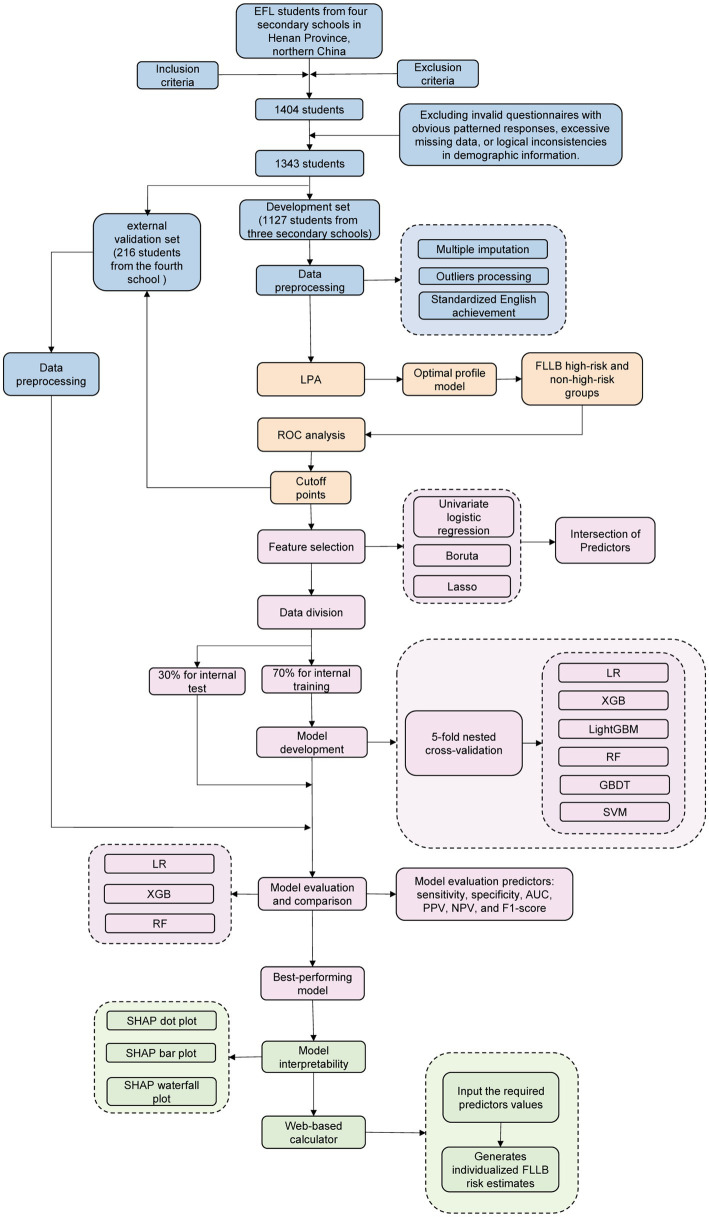
Overall study workflow. EFL, English as a foreign language; LPA, latent profile analysis; ROC analysis, receiver operating characteristic curve analysis; Lasso, least absolute shrinkage and selection operator; LR, logistic regression; XGB, extreme gradient boosting; LightGBM, light gradient boosting machine; RF, random forest; GBDT, gradient boosting decision tree; SVM, support vector machine; AUC, area under receiver-operating curve; PPV, positive predictive value; NPV, negative predictive value; SHAP, Shapley additive explanation.

This study developed an analytical framework for individualized risk identification of FLLB among secondary school students, and the overall workflow is shown in Figure 1. Because FLLB is a multidimensional burnout construct, LPA was first used to identify latent symptom patterns across different burnout dimensions. After the optimal profile model was determined, the FLLB high-risk and non-high-risk groups were defined according to the symptom severity of each profile. ROC analysis was then performed using the high-risk group identified by LPA as the reference classification to determine an operational screening cutoff point. Based on this classification, machine learning models were further developed and validated to estimate the probability that an individual student belonged to the high-risk group. Finally, SHAP analysis was used to visualize the contributions of key predictors, and the final model was deployed as a web-based calculator to improve its feasibility for application in real-world educational settings.

### 2.2 Participants

Using convenience sampling, this study recruited 1,404 EFL students from four secondary schools in Henan Province, northern China, between April 10 and April 25, 2025. Inclusion criteria were: (1) currently enrollment in a secondary school in the province where the study was conducted; (2) voluntary participation and signed informed consent (Liu et al., 2023); for participants younger than 16 years, participation additionally required the individual’s assent together with written parent or legal guardian consent. The exclusion criterion was the presence of severe learning or cognitive difficulties that might interfere with participation (Liu et al., 2023). After excluding 61 questionnaires with obvious patterned responses, excessive missing data, or logical inconsistencies in demographic information (Guan et al., 2025), we had 1,343 valid responses (95.66%). Among these, 1,127 cases from three secondary schools in Henan Province comprised the development set, and 216 cases from another secondary school in Henan Province during the same study period constituted the external validation set. Participants were aged 12 to 19 years, including 690 females and 653 males. During data collection, the questionnaires were anonymized to protect participants’ privacy; after data collection was completed, researchers who were not involved in subsequent data analysis coded the respondents’ names.

Before the survey, we use the “pmsampsize” package in R to conduct a sample size analysis for the development set according to the method proposed by Riley et al. (2020). This package is designed to calculate the minimum sample size required for developing prediction models with continuous, binary, and survival outcomes. The parameter settings included an estimated cstatistic of 0.85, 14 candidate predictors, and a burnout prevalence of 0.217 based on a previous study (Ye et al., 2023). The calculation indicated that the minimum required sample size was 408 participants. The actual sample size of the development set met the requirement for prediction model development. In addition, because no prediction model for FLLB was available in previous studies, this study calculated the sample size for external validation before the survey by referring to the prevalence reported in previous research and assuming at least five events per variable (EPV) (Vittinghoff and McCulloch, 2007). Based on the event proportion of 0.217 reported in previous research and assuming that eight variables would be included after feature selection, at least 40 events were required, corresponding to a minimum sample size of approximately 185 participants. After feature selection was completed, the minimum required sample sizes for the development set and external validation set were recalculated based on the seven variables finally included and the observed prevalence of FLLB in this study, yielding 277 and 246 participants, respectively. The sample size of the development set met the minimum sample size requirement. The sample size of the external validation set was slightly below the recalculated minimum sample size; therefore, it was used only as an exploratory external validation set.

### 2.3 General Information Questionnaire

The General Information Questionnaire included gender (male, female), grade (grade 7-11), class, only child status (only child, non-only child), and family socioeconomic status (including annual family income, parental education level, parental occupation) (Luo et al., 2016). The PSQI comprises 19 items and was developed by Buysse et al. (1989) to assess individual sleep quality; in this study, sleep duration was measured using the fourth item of the PSQI. (Wang F. et al., 2024). English achievement was measured by student’s English score from the citywide unified final exam of the previous semester (Liu H. et al., 2024). The final English exam was designed to assess students’ mastery of the semester’s English curriculum and covered the main components of English learning, including language knowledge, reading, and writing.

### 2.4 Instruments

We administered a general information questionnaire and five well-validated scales assessing foreign language learning burnout, academic stress, engagement, teacher affective support, and foreign language enjoyment. For internal consistency, we calculated Cronbach’s alpha for each scale, interpreting *α* values of 0.9 or above as excellent, 0.7 to 0.9 as satisfactory, 0.6 to 0.7 as acceptable, 0.5 to 0.6 as poor and below 0.5 unacceptable (George and Mallery, 2003).

#### 2.4.1 Foreign language learning burnout

FLLB was evaluated using the scale validated by Li et al. (2024). The scale comprises 10 items across three dimensions: exhaustion (4 items; for example, “English makes me feel mentally exhausted”), cynicism (3 items; for example, “My enthusiasm for learning English has decreased”), and reduced efficacy (3 items; for example, “I do not believe that I can play an active role in English class”). All items are rated on a 5-point Likert scale, with 1 indicating strongly disagree and 5 indicating strongly agree. The Cronbach’s *α* of the scale was 0.886. Given that this study was conducted in China and measured student burnout in an English learning context, this scale was suitable for this study and was therefore adopted. In the present sample, the Cronbach’s *α* of the total scale was 0.948, indicating high internal consistency.

#### 2.4.2 Other factors

Core variables were assessed with validated scales. Academic stress was assessed with the Educational Stress Scale for Adolescents (Sun et al., 2011); The ESSA contains 16 items covering five dimensions: pressure from study, workload, worry about grades, self-expectation, and despondency. The total scale has good internal consistency, with a Cronbach’s *α* of 0.81. Engagement was measured using the Utrecht Work Engagement Scale for Students (Schaufeli et al., 2002), which includes 14 items across three dimensions: vigor, dedication, and absorption. The original study reported Cronbach’s α values of 0.65–0.86 for the three dimensions across samples from different countries, indicating generally acceptable internal consistency. Teacher affective support was evaluated with a scale validated in educational psychology by Sakiz (2007). This nine-item scale captures students’ perceived teacher emotional support, care, respect, and encouragement. In the original study, the scale showed a Cronbach’s α of 0.92 in the preliminary analysis. Foreign language enjoyment was measured using the Chinese version of the Foreign Language Enjoyment Scale (Li et al., 2018). This scale comprises 11 items covering three dimensions: private enjoyment, teacher-related enjoyment, and classroom atmosphere-related enjoyment. The Cronbach’s α of the total scale was 0.826. In the present sample, reliability was excellent across all scales, as indicated by Cronbach’s α coefficients of 0.924, 0.956, 0.960, and 0.932, respectively.

### 2.5 Statistical analysis

All statistical analyses were carried out using R statistics software 4.2.3, Python 3.11.4, and Mplus version 8.3. Descriptive statistics were used to summarize the basic characteristics of the development sample, and univariate analyses were performed to compare differences between the FLLB high-risk and non-high-risk groups. Continuous variables included age, academic stress, engagement, teacher affective support, foreign language enjoyment, English achievement, and sleep duration. Because these variables did not follow a normal distribution (Supplementary File 3), they were described using medians and interquartile ranges (IQR), with group comparisons conducted through the Mann–Whitney U test. Categorical variables included gender, grade, only-child status, family income, parental education level, and parental occupation. Categorical variables were described using numbers and percentages and analyzed using the chi-square test.

Before the main analyses, data preprocessing was performed. The English achievement variable had 29 missing values, corresponding to a missing rate of less than 10%. Therefore, multiple imputation was performed using predictive mean matching (PMM). Boxplots were used to examine outliers in continuous variables (Supplementary File 4). In addition, to improve the potential applicability of the web-based calculator across different schools and regions, the English exam scores were standardized by schools and grades before analysis to indicate each student’s relative standing among within their peer group (Klein, 2014). In the web-based calculator, after users enter the raw English score, the system automatically performs the standardization procedure and uses the standardized score for FLLB risk prediction.

Subsequently, the three dimensions of the FLLB scale were used as indicators, and LPA was performed to identify individual-level heterogeneity in FLLB. Models with one to six profiles were fitted sequentially using Mplus 8.3. Because the data deviated from normality, robust maximum likelihood estimation (MLR) was applied, with the initial and final stage random starts specified as 200 and 50, respectively (Luo et al., 2024). The following indices were considered when determining the optimal model: Akaike Information Criterion (AIC), Bayesian Information Criterion (BIC), the sample size-adjusted BIC (aBIC), the Bootstrap Likelihood Ratio Test (BLRT), the Lo–Mendell–Rubin Test (LMR), and Entropy (Fonseca-Pedrero et al., 2017; Tein et al., 2013). Model fit was assessed using AIC, BIC, and aBIC, with lower values indicating better fit (Zhang et al., 2025). Classification accuracy was assessed using Entropy (0–1), where higher values reflect clearer separation between latent classes (Zhang et al., 2025). Following prior methodological recommendations, we selected entropy values >0.80 (Fonseca-Pedrero et al., 2017). For model comparison, BLRT and LMR tests were performed, and a *p*-value below 0.05 indicated that the model with k profiles fit better than one with k-1 profiles (Luo et al., 2024; Muthén and Muthén, 2017). After the final profile model was determined, students were classified into the FLLB high-risk group and non-high-risk group according to their different levels of FLLB risk. This binary classification was used in the subsequent ROC analysis and machine learning modeling.

Two approaches were used in this study to identify the optimal cutoff point for FLLB: ROC analysis of the total FLLB score and grid search on the basis the three dimension scores. First, on the basis of the high-risk and non-high-risk groups determined by LPA, we performed ROC analysis on the total FLLB score, computed the AUC value, and evaluated sensitivity and specificity for each candidate cutoff (Li et al., 2020); the optimal cutoff point was then selected by the maximum Youden’s index (Akobeng, 2007; Cheng et al., 2025). Subsequently, grid search was performed on the three dimensions of FLLB, exhaustively enumerating all combinations of $\left(x_{1},x_{2},x_{3}\right)$ . Rules were constructed as “Exhaustion ≥ $x_{1}$ and Cynicism ≥ $x_{2}$ and Reduced Efficacy ≥ $x_{3}$ .” For each combination, sensitivity and specificity were obtained from the confusion matrix, and the optimal cutoff point was chosen by maximizing Youden’s index (Akobeng, 2007). Finally, the Youden’s index obtained from the two methods were compared, and confusion matrices were used to visually present the actual classification results of both methods. The method with the higher Youden’s index was selected as the final cutoff point for FLLB and was further applied to the external validation set to classify students into high-risk and non-high-risk FLLB groups.

To identify key predictors of FLLB, this study employed three feature selection methods: Univariate logistic regression, LASSO regression, and the Boruta algorithm (Wan et al., 2025). Univariate logistic regression serves as a conventional screening method, where variables with *p* values below 0.05 were considered statistically significant (Kwiendacz et al., 2025). LASSO regression, a regularization technique, imposes an L1 penalty, shrinking coefficients of irrelevant variables to zero and effectively removing them from the model (Kamkar et al., 2016). Boruta is a machine learning-based feature selection method that identifies features significantly contributing to the target variable by constructing “shadow variables” and comparing their importance (Degenhardt et al., 2019).

### 2.6 Development and evaluation of machine learning models

The development set was randomly split into an internal training set and an internal test set at a 7:3 ratio, and six machine learning algorithms were used to develop and compare diagnostic models for predicting the FLLB risk, including: Logistic Regression, XGBoost, LightGBM, Random Forest, GBDT, and SVM. For hyperparameter optimization, we used 5-fold nested cross-validation along with grid search (Qi et al., 2025) (details in Supplementary File 5). Model evaluation indicators included sensitivity, specificity, area under the receiver operating characteristic curve (AUC), positive predictive value (PPV), negative predictive value (NPV), and F1 score (Hou et al., 2024). Based on these multidimensional indicators, we selected the three best-performing models. These models were then refitted in a 70% internal training set and evaluated for generalization ability in a 30% internal test set and an exploratory external validation set. The model with the most stable generalization ability was selected as the optimal model and interpreted using the SHAP package in Python.^1^ To facilitate practical application, we deployed the final prediction model as an interactive web tool. When users enter the required predictor values, the application returns the risk probability of FLLB and presents both the overall feature contributions of the model and individual-level prediction explanations through SHAP visualizations.

## 3 Results

### 3.1 Descriptive statistics

The development set included 1,127 secondary school students, among whom 160 (14.2%) exhibited foreign language learning burnout (FLLB = 1), while 967 (85.8%) did not (FLLB = 0). There were significant group differences in gender (*p* = 0.05) and grade composition (*p* < 0.05). For continuous variables, significant between-group differences were observed in academic stress (AS), engagement, teacher emotional support (TAS), foreign language enjoyment (FLE), and English achievement (EA) (*p* < 0.05 for all), while sleep duration (SD) showed no significant between-group difference (*p* = 0.3). See Table 1 for details.

**Table 1:** Demographic characteristics of participants in training set.

Characteristic	Overall (*n* = 1,127)	FLLB 1 (*n* = 160)	FLLB 0 (*n* = 967)	Statistics	*P*-value
Gender, *n* (%)	546 (48.45)	89 (55.63)	457 (47.26)	3.85	0.05
	581 (51.55)	71 (44.38)	510 (52.74)		
Grade, *n* (%)	238 (21.12)	32 (20.00)	206 (21.30)	12.77	0.012
	185 (16.42)	26 (16.25)	159 (16.44)		
	189 (16.77)	39 (24.38)	150 (15.51)		
	313 (27.77)	30 (18.75)	283 (29.27)		
	202 (17.92)	33 (20.63)	169 (17.48)		
OC, *n* (%)	46 (4.08)	6 (3.75)	40 (4.14)	0.05	0.819
	1,081 (95.92)	154 (96.25)	927 (95.86)		
FI, *n* (%)	364 (32.30)	55 (34.38)	309 (31.95)	0.87	0.928
	486 (43.12)	66 (41.25)	420 (43.43)		
	172 (15.26)	23 (14.37)	149 (15.41)		
	73 (6.48)	12 (7.50)	61 (6.31)		
	32 (2.84)	4 (2.50)	28 (2.90)		
FE, *n* (%)	121 (10.74)	21 (13.13)	100 (10.34)	1.78	0.776
	744 (66.02)	106 (66.25)	638 (65.98)		
	210 (18.63)	26 (16.25)	184 (19.03)		
	47 (4.17)	6 (3.75)	41 (4.24)		
	5 (0.44)	1 (0.63)	4 (0.41)		
ME, *n* (%)	169 (15.00)	23 (14.37)	146 (15.10)	6.53	0.163
	702 (62.29)	109 (68.13)	593 (61.32)		
	204 (18.10)	23 (14.37)	181 (18.72)		
	46 (4.08)	3 (1.88)	43 (4.45)		
	6 (0.53)	2 (1.25)	4 (0.41)		
FO, *n* (%)	566 (50.22)	84 (52.50)	482 (49.84)	1.66	0.798
	378 (33.54)	48 (30.00)	330 (34.13)		
	117 (10.38)	17 (10.63)	100 (10.34)		
	56 (4.97)	10 (6.25)	46 (4.76)		
	10 (0.89)	1 (0.63)	9 (0.93)		
MO, *n* (%)	762 (67.61)	104 (65.00)	658 (68.05)	2.51	0.643
	167 (14.82)	29 (18.13)	138 (14.27)		
	146 (12.95)	19 (11.88)	127 (13.13)		
	44 (3.90)	6 (3.75)	38 (3.93)		
	8 (0.71)	2 (1.25)	6 (0.62)		
Age, median [IQR]	15 [14, 16]	15 [14, 16]	15 [14, 16]	0.64	0.512
AS, median [IQR]	50 [47, 57]	59 [53, 64]	49 [46, 55]	−10.92	<0.001
Engagement, median [IQR]	43 [42, 50]	42 [38, 45]	43 [42, 51]	4.41	<0.001
TAS, median [IQR]	32 [27, 36]	30 [27, 36]	33 [27, 36]	3.5	<0.001
FLE, median [IQR]	37 [33, 43]	33 [31, 37]	38 [33, 43]	7.35	<0.001
EA, median [IQR]	0.09 [−0.76, 0.84]	−0.33 [−1.06, 0.20]	0.16 [−0.68, 0.88]	5.97	<0.001
SD, median [IQR]	7.8 [7.0, 8.3]	7.5 [7.0, 8.0]	8.0 [7.0, 8.4]	1.02	0.3

OC, only child; FI, family income; FE, father’s education; ME, mother’s education; FO, father’s occupation; MO, mother’s occupation; AS, academic stress; TAS, teacher affective support; FLE, foreign language enjoyment; EA, English achievement; SD, sleep duration; IQR, Interquartile range.

### 3.2 Latent characteristics of foreign language learning burnout

**Figure 2:**
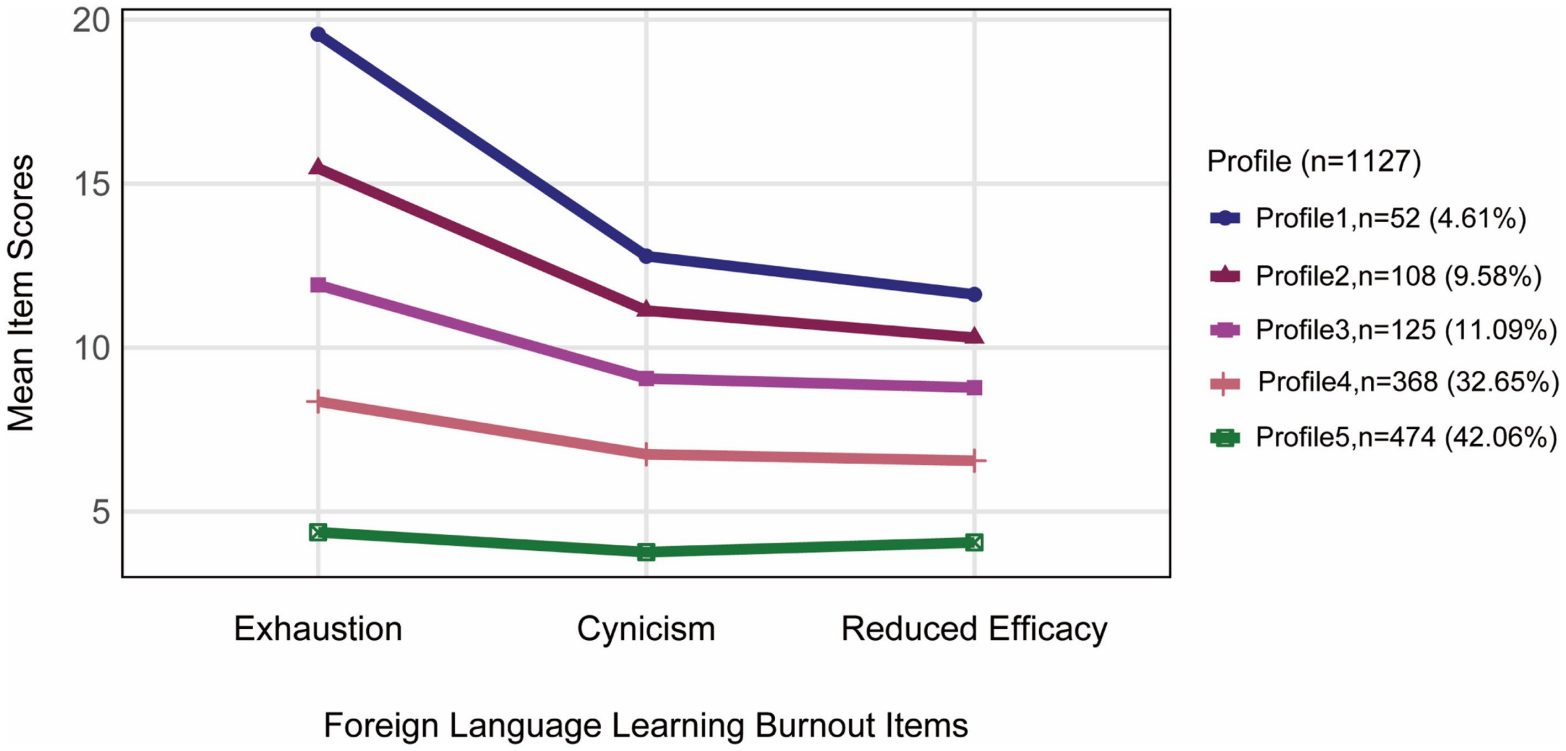
Five profiles of the best-fitting five-class pattern based on the Foreign Language Learning Burnout Scale.

A series of LPA models were estimated, ranging from one to six latent classes, with fit indices summarized in Table 2. As the number of profiles increased, the AIC, BIC, and SABIC values continued to decrease, while entropy gradually increased. However, the pLMR value for the six-profile model was greater than 0.05 and did not reach statistical significance. In addition, this model produced a very small profile comprising only 1.76% of the sample, which may affect classification stability and limit its practical utility in subsequent ROC analysis and predictive modeling. In contrast, the five-profile model showed a better overall fit, with AIC = 14596.098, BIC = 14706.699, SABIC = 14636.821, entropy = 0.954, pLMR = 0.0006, and BLRT < 0.0001. Therefore, after considering the fit indices and substantive interpretability, this study selected the five-profile model as the optimal model for latent profile analysis. Figure 2 depicts the five-profile model of FLLB. Because Profile 1 accounted for 4.6% of the sample, which was slightly below the 5% empirical criterion used in previous studies to judge the feasibility of class solutions, each latent profile should include at least 5% of the total sample to exclude substantively unreasonable class solutions and reduce the risk of overfitting (Wendt et al., 2019). In addition, Profile 1 and Profile 2 both showed relatively high burnout characteristics across the three dimensions of exhaustion, cynicism, and reduced efficacy. Therefore, we combined Profiles 1 and 2 and defined them as the foreign language learning burnout group (FLLB = 1). The remaining Profiles 3–5 were defined as the non-burnout group (FLLB = 0). This classification strategy considered profile symptom severity, classification interpretability and stability, and the operational feasibility of subsequent screening and predictive modeling. Based on this, we first conducted ROC analysis on the FLLB total scores, yielding an optimal cutoff point of 33.5 (the Youden’s index = 0.913, sensitivity = 0.938, and specificity = 0.975). Results are presented in Supplementary File 6. We then performed an exhaustive grid search over threshold combinations for the three learning burnout dimensions and evaluated the classification performance for each possible score combination. The optimal combination was: Exhaustion ≥14, Cynicism ≥3, and Reduced Efficacy ≥3 (the Youden’s index = 0.982, sensitivity = 1.000, and specificity = 0.982). Results are presented in Supplementary File 7. By comparing the maximum Youden index values obtained from the two methods and using confusion matrices to visually present the actual classification results of both methods (Supplementary File 8), the combination of Exhaustion ≥14, Cynicism ≥3, and Reduced Efficacy ≥3 was ultimately determined as the optimal cutoff point. This cutoff point was subsequently applied to the exploratory external validation set to stratify populations at risk for FLLB (details in Supplementary File 9).

**Table 2:** Fit statistics for the latent profile analysis and the corresponding profile probability.

Classes	LogLik	AIC	BIC	SABIC	Entropy	pLMR	pBLRT	Profile_Prob
1	−8493.818	16999.637	17029.801	17010.743				
2	−7900.176	15820.353	15870.626	15838.863	0.838	0	0	40.98/59.02
3	−7619.392	15266.783	15337.166	15292.698	0.874	0	0	53.95/36.20/9.85
4	−7383.544	14803.089	14893.58	14836.407	0.901	0	0	33.01/10.12/47.21/9.67
5	−7276.049	14596.098	14706.699	14636.821	0.954	0.0006	0	32.65/11.09/42.06/4.61/9.58
6	−7234.049	14520.098	14650.808	14568.225	0.956	0.0647	0	1.76/10.20/32.12/41.62/4.61/9.67

AIC, Akaike information criterion; BIC, Bayesian information criterion; SABIC, sample-size adjusted BIC; BLRT, bootstrap likelihood ratio test; LMR, Lo–Mendell–Rubin test.

### 3.3 Predictors of foreign language learning burnout

**Figure 3:**
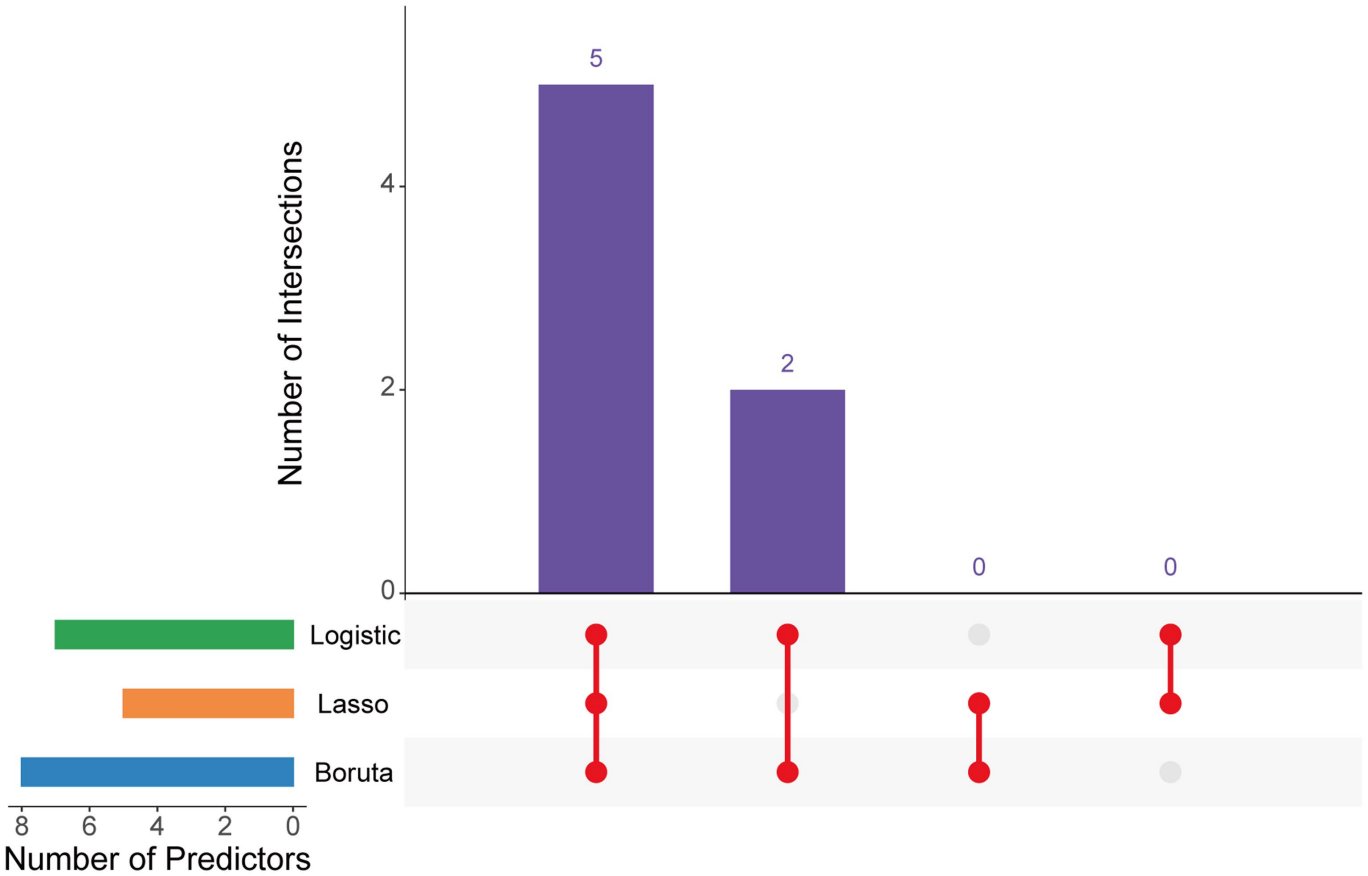
Upset plot of interactions between the predictors. Horizontal bars indicate the number of variables for each method individually, and vertical bars indicate mutually exclusive intersections of different combinations of methods: two-by-two intersections exclude variables that have been counted in the intersection of the three. A bar height of 0 indicates that the variables common to that combination are already included in the higher-order intersection and will not be counted again. Red dots and lines mark the methods that participate in the intersection. Logistic univariate logistic analysis, LASSO least absolute shrinkage and selection operator.

Multiple feature selection methods were used to identify key predictors of FLLB risk. Univariate logistic regression screened seven variables; the Boruta algorithm confirmed eight variables; LASSO identified five variables, as detailed in Supplementary File 10. To synthesize results across methods and maximize retention of stable, predictive variables, we followed prior research (Qi et al., 2025) and computed pairwise intersection among the three methods’ outputs, as shown in Figure 3. Seven variables were chosen as input features in subsequent model development: gender, grade, academic stress, engagement, teacher affective support, foreign language enjoyment, and English achievement.

### 3.4 Comparison of multiple classification models

**Figure 4:**
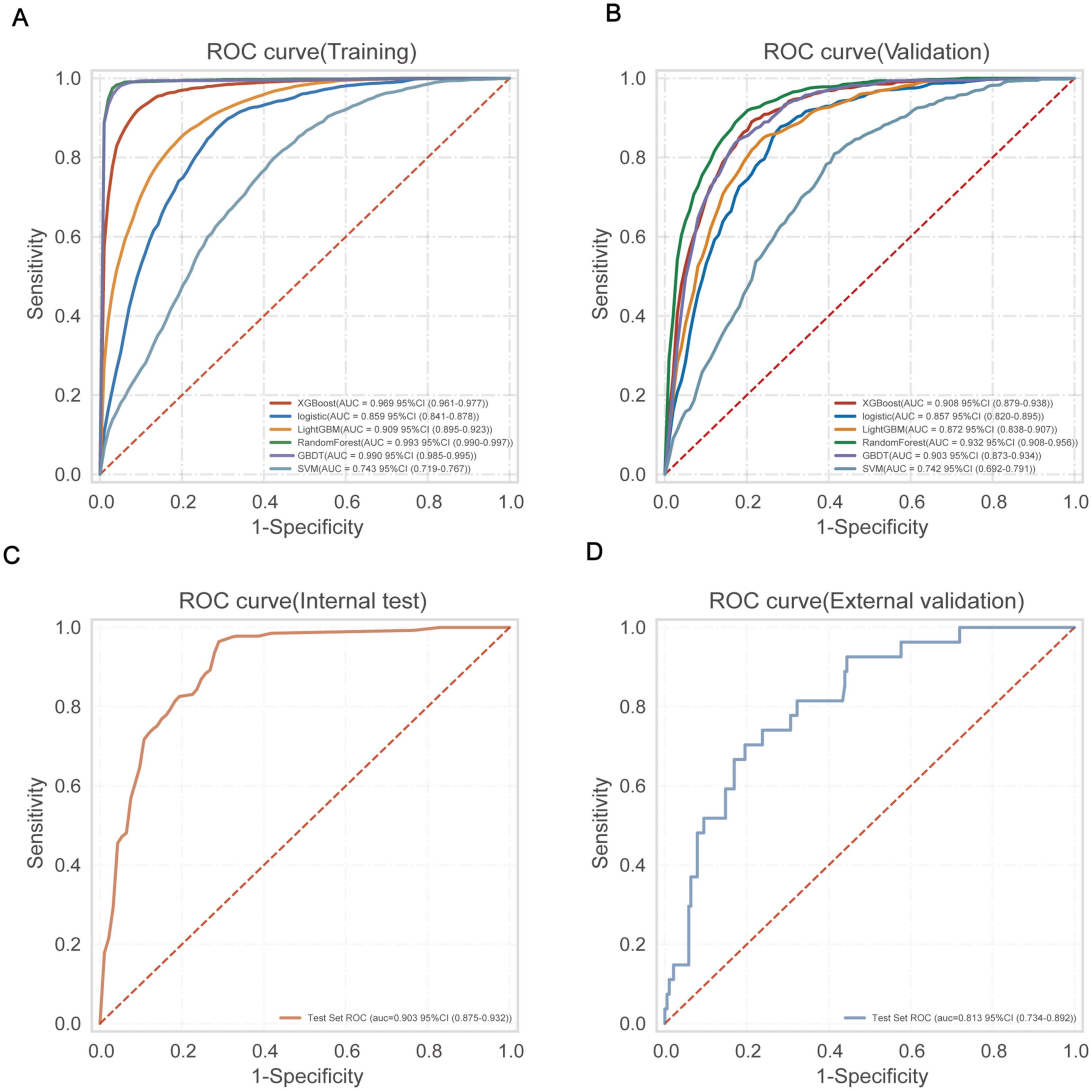
Predictive performance of six machine learning models and logistic regression on foreign language learning burnout. **(A,B)** ROC curves of six machine learning algorithms in the training and validation sets. **(C,D)** ROC curves of the logistic regression model in the internal test set and external validation set.

Each of the six models were first optimized with its respective optimal hyperparameter settings. As shown in Table 3, their performance was then compared in the training and validation sets. In the training set, Random Forest (AUC = 0.993), GBDT (AUC = 0.990), and XGBoost (AUC = 0.969) demonstrated relatively high discriminatory abilities. Logistic Regression followed with AUC values 0.859. However, in the validation set, the discriminatory performance of Random Forest, GBDT, XGBoost, all decreased, with AUC values of 0.932, 0.908, and 0.903, whereas Logistic Regression remained at 0.857. Results are shown in Figures 4A,B. Overall, Random Forest performed best in the training set, XGBoost demonstrated stability while maintaining high discriminatory ability, and Logistic Regression showed good stability across different datasets.

**Table 3:** Prediction performance metrics of six machine learning algorithms in training and validation sets.

Models	AUC (95%CI)	Cutoff (95%CI)	Accuracy (95%CI)	Sensitivity (95%CI)	Specificity (95%CI)	PPV (95%CI)	NPV (95%CI)	F1 score (95%CI)	Kappa (95%CI)
Training set									
XGBoost	0.969 (0.961–0.977)	0.536(0.518–0.555)	0.916(0.910–0.922)	0.931(0.921–0.940)	0.901(0.880–0.922)	0.904(0.887–0.922)	0.929(0.921–0.936)	0.917(0.912–0.922)	0.832(0.819–0.844)
LR	0.859 (0.841–0.878)	0.472(0.456–0.488)	0.797(0.794–0.801)	0.881(0.861–0.902)	0.713(0.693–0.734)	0.755(0.745–0.764)	0.858(0.841–0.876)	0.813(0.808–0.818)	0.595(0.588–0.602)
LightGBM	0.909 (0.895–0.923)	0.503(0.501–0.506)	0.827(0.820–0.834)	0.829(0.801–0.857)	0.825(0.802–0.847)	0.826(0.812–0.841)	0.829(0.809–0.850)	0.827(0.818–0.836)	0.654(0.641–0.667)
RF	0.993 (0.990–0.997)	0.574(0.529–0.618)	0.971(0.965–0.977)	0.973(0.962–0.983)	0.969(0.960–0.978)	0.969(0.961–0.978)	0.973(0.963–0.983)	0.971(0.965–0.977)	0.942(0.930–0.953)
GBDT	0.990 (0.985–0.995)	0.908(0.730–1.087)	0.956(0.947–0.965)	0.937(0.910–0.965)	0.975(0.963–0.986)	0.974(0.963–0.985)	0.94(0.916–0.964)	0.955(0.945–0.965)	0.912(0.894–0.929)
SVM	0.743 (0.719–0.767)	0.526(0.447–0.606)	0.691(0.667–0.716)	0.77(0.694–0.846)	0.613(0.549–0.677)	0.667(0.641–0.693)	0.733(0.683–0.783)	0.712(0.679–0.745)	0.382(0.333–0.431)
Validation set									
XGBoost	0.908 (0.879–0.938)	0.536(0.518–0.555)	0.831(0.819–0.843)	0.845(0.824–0.865)	0.817(0.776–0.857)	0.823(0.794–0.853)	0.841(0.828–0.854)	0.833(0.825–0.841)	0.662(0.638–0.685)
LR	0.857 (0.820–0.895)	0.472(0.456–0.488)	0.797(0.786–0.807)	0.881(0.862–0.899)	0.712(0.692–0.733)	0.754(0.742–0.766)	0.857(0.840–0.874)	0.812(0.803–0.822)	0.593(0.573–0.613)
LightGBM	0.872 (0.838–0.907)	0.503(0.501–0.506)	0.795(0.771–0.818)	0.792(0.763–0.821)	0.797(0.731–0.863)	0.8(0.755–0.845)	0.794(0.780–0.808)	0.794(0.778–0.811)	0.589(0.542–0.636)
RF	0.932 (0.908–0.956)	0.574(0.529–0.618)	0.855(0.847–0.862)	0.865(0.834–0.897)	0.844(0.807–0.880)	0.849(0.822–0.875)	0.864(0.840–0.888)	0.856(0.848–0.864)	0.709(0.694–0.725)
GBDT	0.903 (0.873–0.934)	0.908(0.730–1.087)	0.789(0.754–0.824)	0.704(0.580–0.828)	0.874(0.811–0.936)	0.858(0.814–0.901)	0.757(0.693–0.822)	0.763(0.698–0.828)	0.578(0.508–0.647)
SVM	0.742 (0.692–0.791)	0.526(0.447–0.606)	0.685(0.669–0.701)	0.764(0.696–0.831)	0.607(0.563–0.650)	0.66(0.651–0.670)	0.726(0.679–0.773)	0.707(0.678–0.735)	0.37(0.338–0.403)

XGB, extreme gradient boosting; LR, logistic regression; LightGBM, Light Gradient Boosting Machine; RF, random forest; GBDT, Gradient Boosting Decision Tree; SVM, support vector machine; AUC, area under receiver-operating curve; PPV, positive predictive value; NPV, negative predictive value.

Based on the overall performance and characteristics of the models trained, this study selected Random Forest, XGBoost, and Logistic Regression for further evaluation of their generalization abilities. As shown in Table 4, the three models showed comparable discrimination in the internal test set. Logistic Regression achieved an AUC of 0.903, followed by Random Forest with an AUC of 0.902 and XGBoost with an AUC of 0.899, with Logistic Regression performing slightly better. In the exploratory external validation set, both Random Forest and XGBoost exhibited notable declines in performance, suggesting a degree of overfitting. In contrast, Logistic Regression maintained relatively stable performance with an AUC of 0.813, as illustrated in Figures 4C,D.

**Table 4:** Predictive performance metrics of logistic regression in the internal test set and external validation set.

Models	AUC (95%CI)	Cutoff	Accuracy	Sensitivity	Specificity	PPV	NPV	F1 score	Kappa
Internal test set									
LR	0.903 (0.875–0.932)	0.517	0.797	0.978	0.712	0.615	0.986	0.755	0.597
XGBoost	0.899 (0.870–0.928)	0.593	0.818	0.796	0.829	0.686	0.896	0.736	0.599
RF	0.902 (0.869–0.934)	0.56	0.839	0.847	0.836	0.707	0.921	0.771	0.648
External validation set									
LR	0.813(0.734–0.892)	0.517	0.694	0.815	0.677	0.265	0.962	0.4	0.261
XGBoost	0.735(0.651–0.819)	0.593	0.769	0.444	0.815	0.255	0.911	0.324	0.197
RF	0.744(0.652–0.836)	0.56	0.759	0.556	0.788	0.273	0.925	0.366	0.238

Overall, although XGBoost and Random Forest showed higher discrimination during model development, this advantage was not maintained in the exploratory external validation set. In contrast, Logistic Regression showed more stable performance between the internal test set and the exploratory external validation set, while maintaining relatively good discriminative ability. Therefore, we selected logistic regression as the final model and used it for subsequent SHAP-based interpretation and web calculator deployment. So, we selected Logistic Regression as the final model. It was then used for further interpretative analysis and deployed in as the web-based calculator.

### 3.5 Interpretability of the optimal model

**Figure 5:**
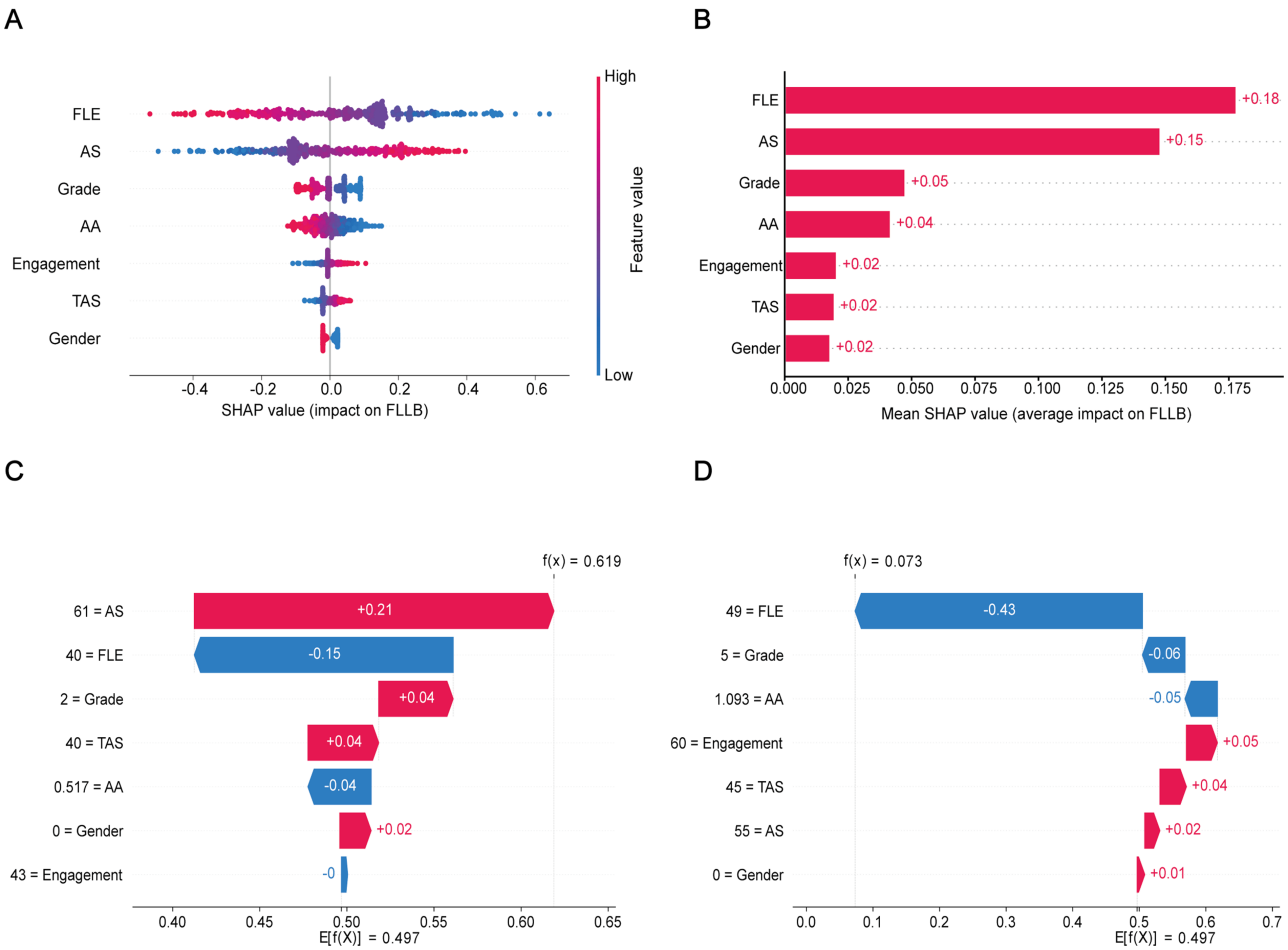
The feature importance and interpretation of the model by the SHapley Additive exPlanation (SHAP) method. **(A)** A summary plot of the SHAP values for each feature, **(B)** the importance ranking of features based on the mean (|SHAP value|), **(C,D)** SHAP waterfall plot.

To enhance model transparency, we use the SHAP method to interpret the final logistic regression model. Figure 5A gives the SHAP summary dot plot, in which the orientation and intensity of the effect of each predictor on high FLLB risk can be visualized clearly. In the figure, higher foreign language enjoyment scores indicate lower FLLB risk, while higher academic stress levels have been found to correlate with higher FLLB risk. In the SHAP summary bar plot (Figure 5B), in which features are ranked based on mean SHAP values, foreign language enjoyment and academic stress emerge as the two most influential predictors of FLLB. At the individual level, the SHAP waterfall plots (Figures 5C,D) clearly illustrate how specific features jointly influenced the predicted risk for each sample. For example, in Figure 5C, high academic stress combined with low foreign language enjoyment strongly drives the prediction toward high risk; although academic achievement exerted a modest protective effect, its influence was insufficient to offset the overall tendency toward high risk.

### 3.6 Implementation of the web-based calculator

**Figure 6:**
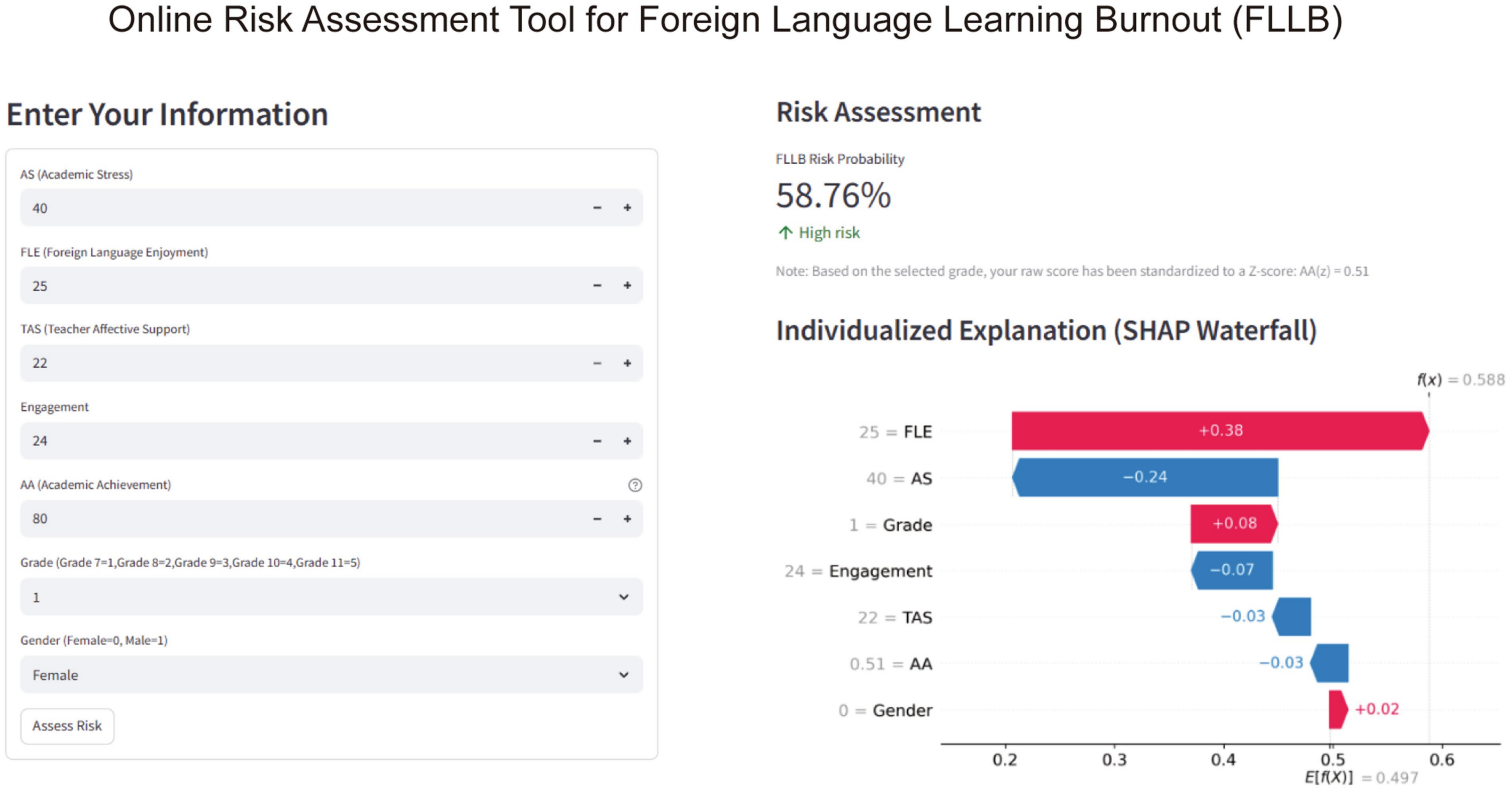
The web-based calculator for predicting the risk of foreign language learning burnout among secondary school students.

We developed a web-based application for individualized FLLB risk assessments on the basis of the final model, as shown in Figure 6. After the values of the seven required predictors are entered, the application automatically generates the estimated risk of FLLB for each student. This web-based tool is accessible online at the following links: https://fllb-shap.streamlit.app/.

## 4 Discussion

This study pioneers the integration of LPA and ROC analysis to establish an operational cutoff point for assessing FLLB among Chinese secondary school students. This finding addresses the longstanding absence of a unified classification standard for FLLB. Additionally, we developed a machine learning prediction model and employed SHAP values to interpret the model, thereby revealing key predictors and characterizing the direction and relative importance of their effects. Building on preliminary screening with the optimal cutoff point, this study deployed the best-performing machine learning model as a web-based calculator that generates clear prediction outputs to support practical FLLB risk screening and assessment.

### 4.1 Determination of foreign language learning burnout profiles and the optimal cutoff point: LPA and ROC analysis

After identifying five profiles of FLLB through LPA, this study merged Profile 1 and Profile 2 into a high-risk group. Results indicated that 14.2% of students belonged to the high-risk FLLB group, a proportion comparable to burnout prevalence reported in previous studies (Salmela-Aro et al., 2016; Vansoeterstede et al., 2024), suggesting that FLLB has become a widespread and noteworthy educational concern. However, previous LPA-based studies have mostly reported only the number and characteristics of latent classes (Cano et al., 2024; Tuominen-Soini and Salmela-Aro, 2014), with few efforts to translate profile results into actionable cutoff points for burnout screening. To address this limitation, this study attempted to integrate the high-risk classification derived from LPA with ROC analysis to establish an operational cutoff point for preliminary risk identification. Specifically, individuals were classified as high risk for FLLB when exhaustion ≥14, cynicism ≥3, and reduced efficacy ≥3; Compared with previous studies that used only total scale scores as classification cutoff point (Luo et al., 2025; Peng et al., 2023), the three-dimensional cutoff point proposed in this study showed high discriminative performance in identifying high-risk students, with a Youden’s index of 0.982, sensitivity of 1.000, and specificity of 0.982. This result may be related to the multidimensional structure of burnout. Previous research has suggested that burnout consists of exhaustion, cynicism, and reduced efficacy, with exhaustion occupying a central position among the three dimensions of burnout (Wang Y. et al., 2024), and that different combinations of high and low levels across these three dimensions may form distinct burnout patterns (Leiter and Maslach, 2016). Therefore, the three-dimensional cutoff point may better capture the symptom configuration of high-risk individuals across the core burnout dimensions, whereas a single total score may obscure individual differences reflected by different dimensional combinations.

### 4.2 Individualized risk prediction for foreign language learning burnout: model development, interpretation, and application

Existing research on FLLB primarily examine relationships among variables using regression or structural equation modeling (Cong et al., 2024; Zheng et al., 2024). While informative about population-level patterns, these approaches are not readily suited to precise, individual risk prediction. Against this backdrop, this study is among the first to use machine learning algorithms for the development of a simple, user-friendly model to predict FLLB risk among secondary school students.

During model development, we compared six machine learning algorithms and controlled overfitting through nested cross-validation. Based on overall performance we selected logistic regression as the final model. In the external validation set, it achieved an AUC of 0.813, demonstrating good generalization ability and stability. Although no prior machine learning prediction studies exist specifically for FLLB, related research has been conducted elsewhere. For example, Feher et al. (2024) constructed prediction models in teachers and achieved a discrimination performance of 0.811 in an internal holdout test set. However, this study lacked independent external validation, suggesting that although machine learning demonstrates feasibility in predicting burnout, it remains insufficient for practical applications. In contrast, after comparing multiple machine learning algorithms, this study further evaluated the performance of the final model in an exploratory external validation set. The results supported the robustness of the model and suggested its potential application value in secondary school foreign language education settings.

This study further enhances model interpretability to make the results more accessible in practice. Existing machine learning studies on burnout prediction primarily focuses on overall feature importance analysis and seldom provides explanations at the individual level. For example, Van Zyl-Cillié et al. (2024) highlighted the significance of fatigue and managerial support in the nursing sample but did not provide an interpretative framework at an individual level. In this study, SHAP enables both perspectives: it ranks the overall influence of variables such as academic stress on burnout risk and, at the individual level, it identified specific factors that elevates a particular student’s risk. Furthermore, we deployed the final model as a web-based calculator. Combine with the determined cutoff point, this tool generates clear risk predictions and may facilitate practical FLLB risk screening and assessment.

### 4.3 Risk and protective factors for foreign language learning burnout: evidence within the JD–R framework

The main predictors identified in this study can be interpreted within the demands–resources framework of the JD–R model. Specifically, high demands like heavy academic stress significantly elevate FLLB risk, whereas resource factors, such as teacher affective support, appear to protect against burnout. Among demand variables, academic stress emerged as a key predictor of FLLB. Consistent with prior research (Gao, 2023; Xu et al., 2022), the results of this study showed that academic stress was significantly and positively correlated with FLLB. Academic stress may affect burnout both directly and indirectly, elevating burnout risk through psychological processes such as heightened anxiety and reduced academic self-efficacy (Gao, 2023; Jung et al., 2015). Regarding resource variables, this study identified teacher affective support as significantly and negatively predicting FLLB. Language learning is inherently highly interactive (Jiang et al., 2021), therefore, the emotional support provided by teachers in the classroom may represent an important learning resource for students. Positive teacher-student relationships and an emotionally supportive classroom atmosphere are not only directly associated with lower burnout but may also contribute to burnout indirectly through students’ intrinsic motivation and shame (Karimi and Fallah, 2021).

Higher student engagement was also negatively related to FLLB, consistent with the latent profile analysis findings of Wu et al. (2024b4). From the JD–R theoretical perspective, academic engagement typically represents a positive state arising from ample resources (Bakker and Mostert, 2024). Students with high engagement tend to have stronger intrinsic motivation and external support (Zhang and Hu, 2025), which may make them more resilient against burnout (Kuokkanen et al., 2024; Wang X. C. et al., 2024). Foreign language enjoyment likewise significantly reduced FLLB risk. This accords with findings among Chinese EFL learners (Song, 2024) suggesting that positive learning experiences may constitute crucial protective resources against burnout. Such a protective role may be explained by the capacity of positive emotions to foster psychological resilience and improve students’ ability to manage pressure, which in turn may alleviate burnout (Gong et al., 2023; Song, 2024). Additionally, the results of this study showed that English achievement predicted FLLB, consistent with longitudinal evidence (Paloș et al., 2019) indicating higher academic achievement predicts lower subsequent burnout. Given the exam-oriented nature of English education in China, students often bear the pressure to achieve high scores in order to satisfy expectations from significant others and maintain self-identity (Li et al., 2024). Under such conditions, exam scores are more closely associated with outcome-focused academic emotions such as burnout.

Our findings also indicate that demographic characteristics such as gender and grade contributed to prediction, suggesting that these factors should not be overlooked in risk identification. Prior evidence on gender differences has been mixed (Bikar et al., 2018; Cadime et al., 2016; Salmela-Aro et al., 2008; Vinter et al., 2021), indicating that gender differences in burnout may be influenced by cultural contexts, measurement dimensions, and sample characteristics. This study further showed greater FLLB risk at higher grade levels, consistent with prior adolescent studies (Liu W. et al., 2024; Zhang et al., 2024). This likely reflects heavier course loads and increased examination pressure faced by senior students, which may translate into higher academic stress and emotional burdens (Wuthrich et al., 2020). Anxiety, depression, and other internalizing symptoms induced by these pressures may partially mediate this association, exacerbating burnout (Liu W. et al., 2024; Steare et al., 2023).

### 4.4 Limitations and future directions

We recognize this study has several limitations. First, due to the cross-sectional design, definitive causal relationships among the variables are precluded. In addition, the convenience sampling method used in this study may have introduced selection bias. Therefore, future longitudinal studies with larger and more representative samples are needed to examine the robustness of the findings and the direction of the causal relationships. Second, although this study adopted a relatively systematic data preprocessing procedure and ultimately selected logistic regression as a classifier with relatively stable performance and generalizability, most variables were still derived from self-reported data collected in this cross-sectional survey. Therefore, the findings may be affected by common method variance. This may influence the strength of associations among variables and the estimation of the relative importance of predictors in the model. Future studies should incorporate multisource data to further validate the key predictors identified by the model in this study. Third, there is currently no widely accepted independent gold standard for diagnosing FLLB. In this context, this study identified high-risk profiles using LPA and further determined an operational cutoff point through ROC analysis. Because this cutoff point was derived from a data-driven reference classification, the results should be interpreted as a preliminary screening cutoff point for identifying students at high risk of FLLB rather than as a diagnostic criterion. Future studies should further examine the stability and applicability of this cutoff point in independent samples by incorporating external criteria, professional educational psychological assessments, or longitudinal outcomes. Fourth, the sample in this study consisted of secondary school students from the same region of China. Therefore, caution is needed when applying the cutoff point and prediction model proposed in this study to other populations and contexts. Finally, although we used cross-validation to mitigate overfitting, the model’s practical applicability and operational feasibility in real-world settings still require further evaluation.

Overall, the multidimensional FLLB cutoff point and web-based calculator proposed in this study may provide a useful reference for preliminary identification, stratified screening, and subsequent support and decision-making for FLLB risk in settings where psychological assessment resources are relatively limited. Future studies could integrate this predictive model into routine screening in the form of an online calculator and assess its effectiveness and usability in practice; at the same time, causal-inference methods and intervention studies would be valuable for further establishing the model’s practical relevance from an educational psychology perspective and for exploring individualized intervention strategies.

## 5 Conclusion

This study proposed the optimal cutoff point for FLLB. And on this basis, a prediction model was developed and validated. We further deployed the prediction model as a web-based calculator, thereby achieving effective translation of research findings into practical application. Based on the established cutoff point, this tool enables efficient preliminary screening for FLLB risk, helping schools identify learners at risk of burnout earlier in foreign language learning contexts and providing a reference for subsequent learning support and psychoeducational services. Overall, this study established a comprehensive workflow, from the optimal cutoff point and interpretable prediction models to tool deployment, providing a substantive reference for FLLB risk assessing and screening in real-world settings.

## Data Availability

The raw data supporting the conclusions of this article will be made available by the authors, without undue reservation.

## Footnotes

1
https://shap.readthedocs.io/en/latest/
