# Supplementary material for: Identifying foreign language learning burnout: latent profiles, cutoff points, and an explainable web-based calculator
Source: Front Psychol. 2026 Jun 17;17:1836626. doi: 10.3389/fpsyg.2026.1836626 (PMC13318977; doi:10.3389/fpsyg.2026.1836626)
Supplement: Supplementary file 1 [file Data_Sheet_1.zip › Supplementary Files/Supplementary File 10.pdf]

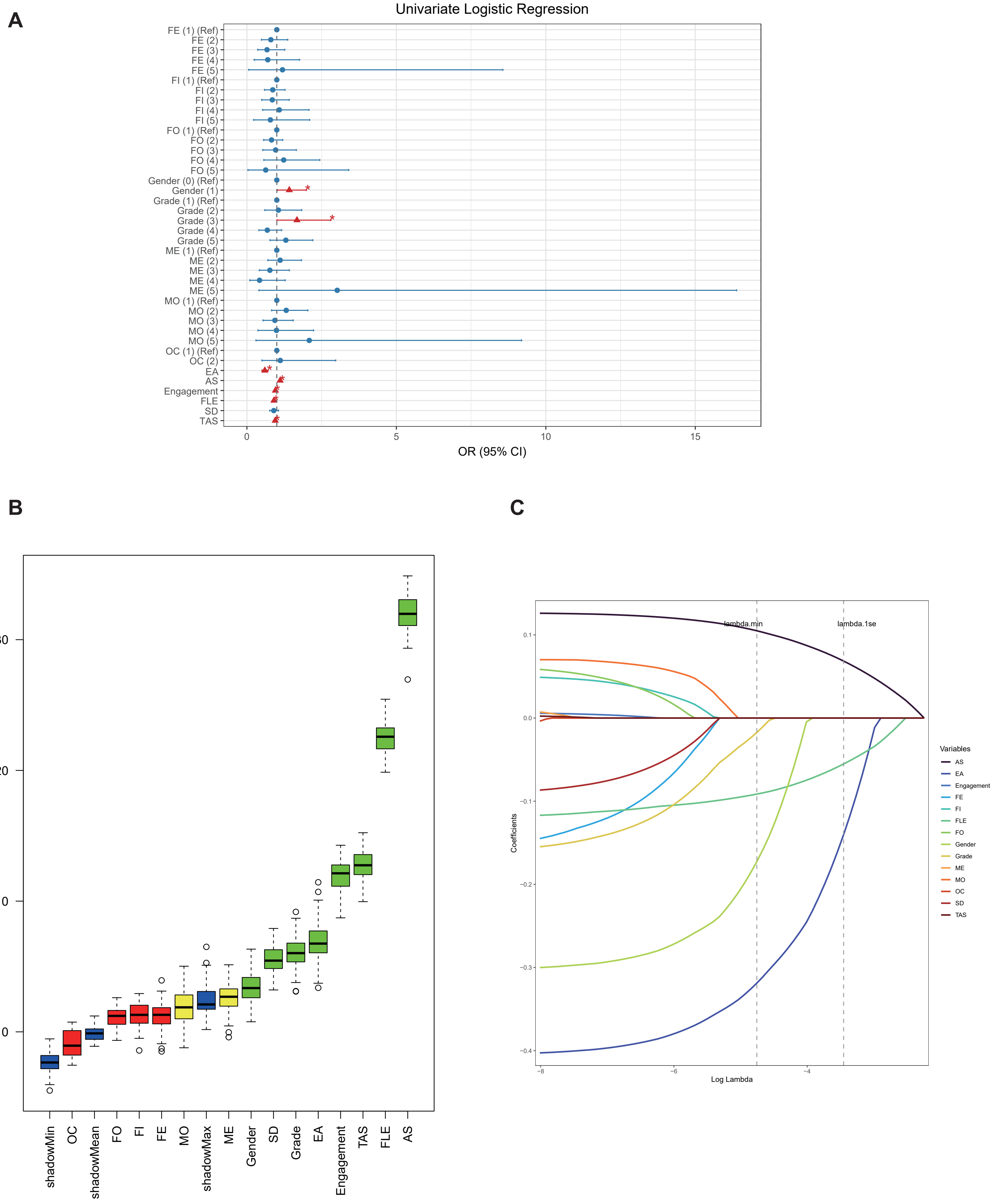

Supplementary File 6: Feature selection results from univariate logistic regression (A), Boruta (B), and LASSO (C)
