# Supplementary material for: Identifying foreign language learning burnout: latent profiles, cutoff points, and an explainable web-based calculator
Source: Front Psychol. 2026 Jun 17;17:1836626. doi: 10.3389/fpsyg.2026.1836626 (PMC13318977; doi:10.3389/fpsyg.2026.1836626)
Supplement: Supplementary file 1 [file Data_Sheet_1.zip › Supplementary Files/Supplementary File 4.pdf]

A

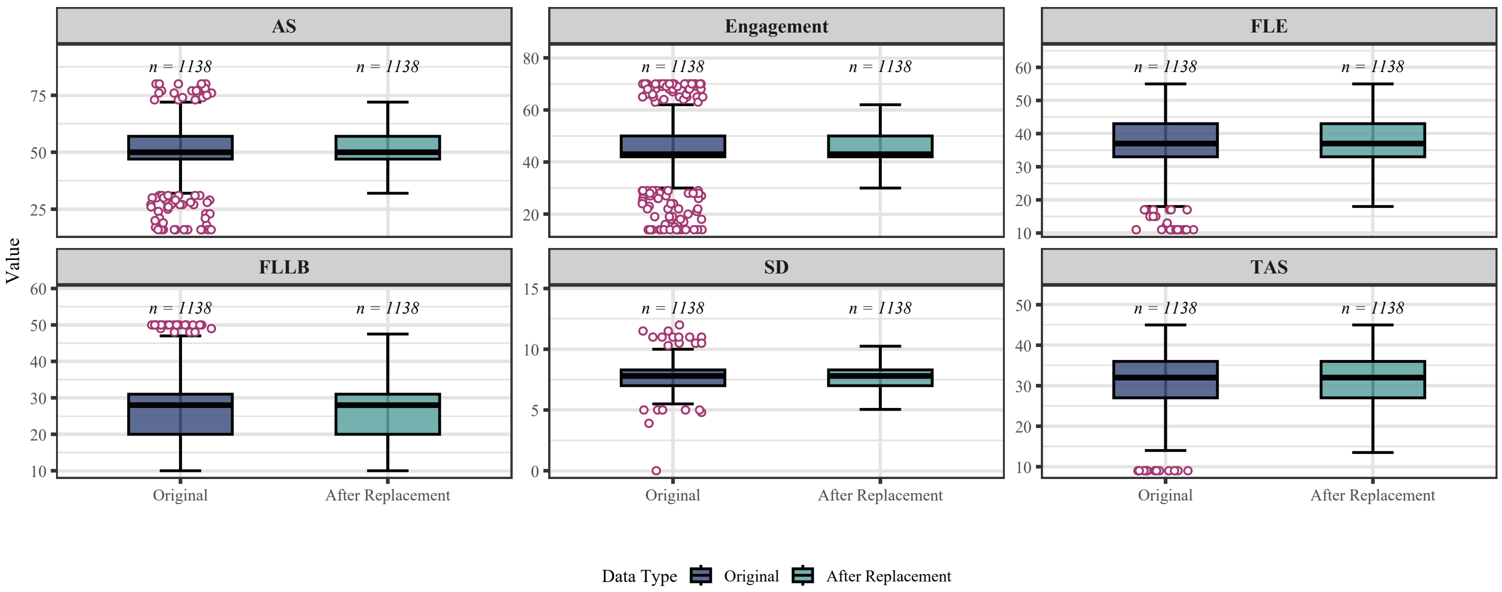

B

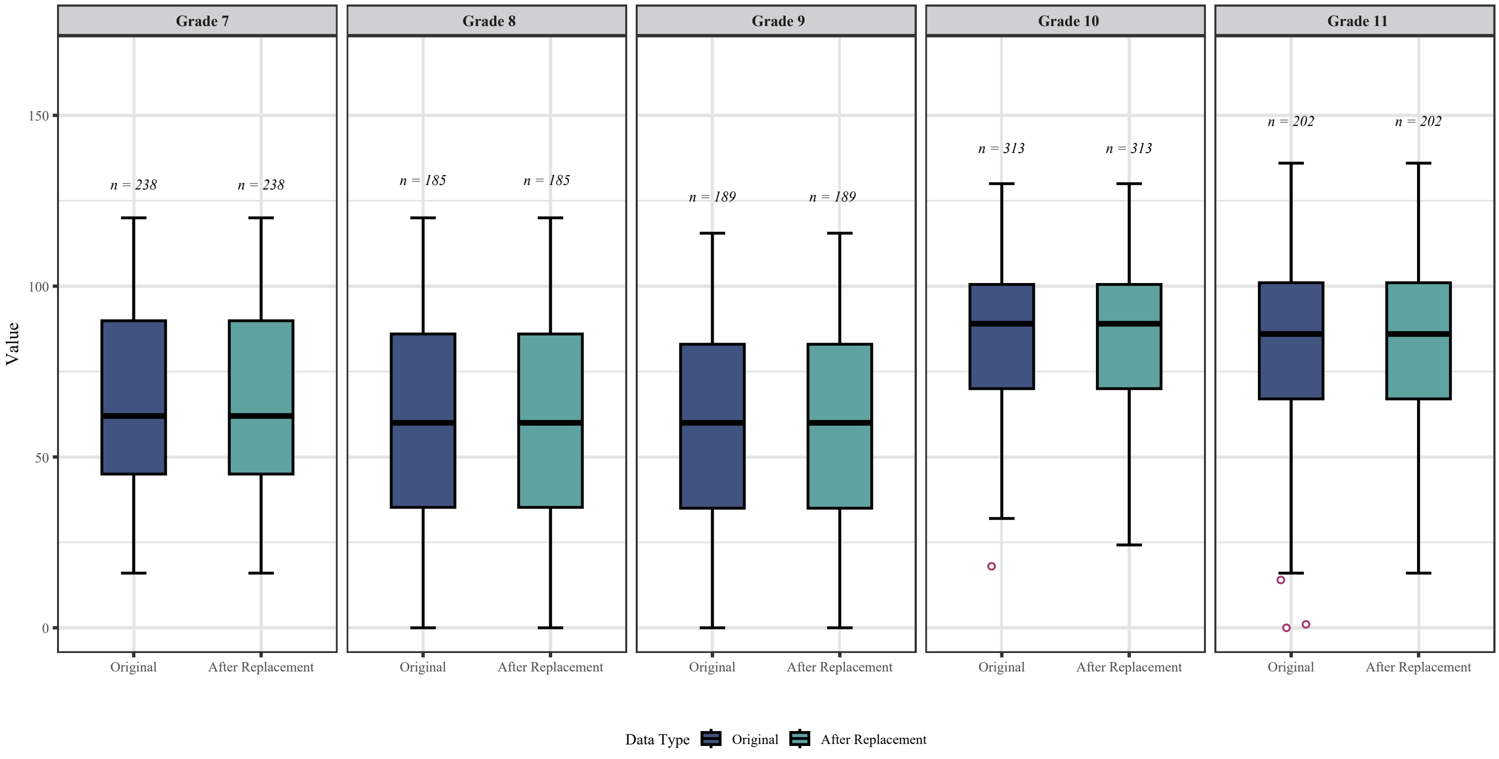

Supplementary File 2: Comparison of the original outliers and their replaced counterparts in continuous variables. The boxplots depict the distribution of each variable before and after processing. (A) Boxplots of academic stress (AS), engagement, foreign language enjoyment (FLE), foreign language learning burnout (FLLB), sleep duration (SD), and teacher affective support (TAS) scores before and after outlier handling. (B) Boxplots of academic achievement scores from Grade 7 to Grade 11 before and after outlier handling.
