# Supplementary material for: Identifying foreign language learning burnout: latent profiles, cutoff points, and an explainable web-based calculator
Source: Front Psychol. 2026 Jun 17;17:1836626. doi: 10.3389/fpsyg.2026.1836626 (PMC13318977; doi:10.3389/fpsyg.2026.1836626)
Supplement: Supplementary file 1 [file Data_Sheet_1.zip › Supplementary Files/Supplementary File 8.pdf]

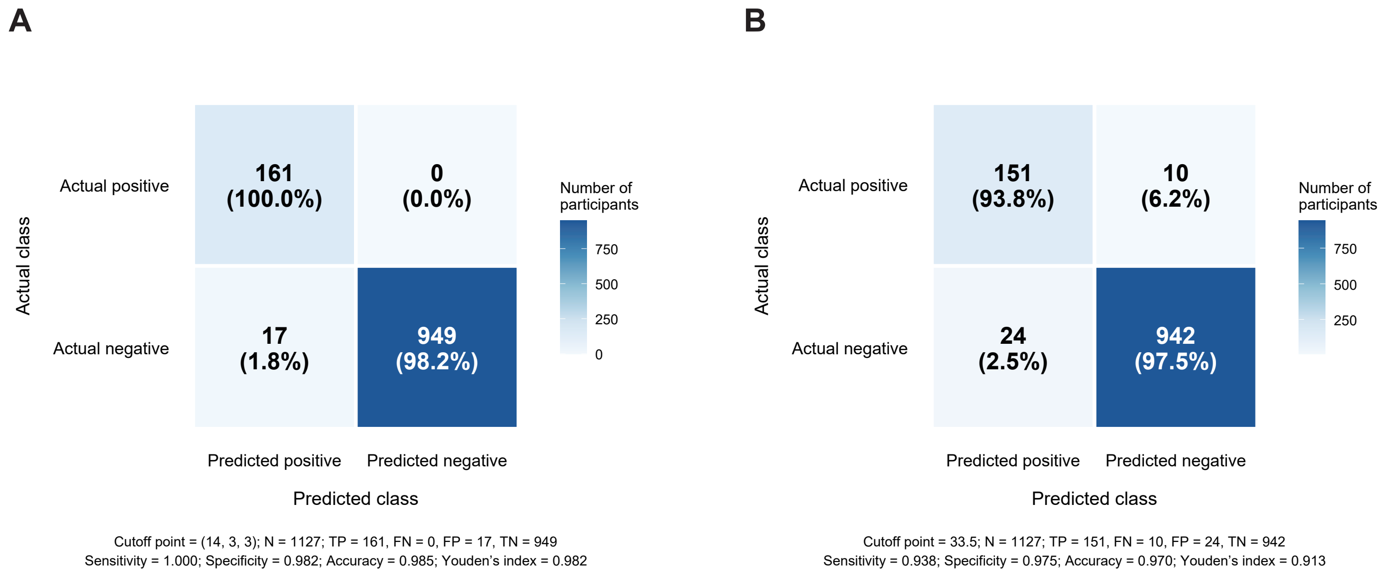

Supplementary File 8: Confusion matrices for FLLB risk classification using the total-score cutoff point (A) and the three-dimensional cutoff point (B)
